# A case of disseminated Legionnaires’ disease: The value of metagenome next-generation sequencing in the diagnosis of Legionnaires

**DOI:** 10.3389/fmed.2022.955955

**Published:** 2022-09-26

**Authors:** Shan Li, Wei Jiang, Chun-Yao Wang, Li Weng, Bin Du, Jin-Min Peng

**Affiliations:** Medical Intensive Care Unit, Peking Union Medical College Hospital, Chinese Academy of Medical Sciences, Beijing, China

**Keywords:** disseminated Legionnaires’ disease, metagenome next-generation sequencing, hospital-acquired pneumonia, immunocompromised adult, blood

## Abstract

**Background:**

*Legionella* rarely causes hospital-acquired pneumonia (HAP), although it is one of the most common pathogens of community-acquired pneumonia. Hospital-acquired Legionnaires’ disease, mainly occurring in immunocompromised patients, is often delayed in diagnosis with high mortality. The use of the metagenome Next-Generation Sequencing (mNGS) method, which is fast and unbiased, allows for the early detection and identification of microorganisms using a culture-independent strategy.

**Case report:**

A 52-year-old male, with a past medical history of Goods syndrome, was admitted due to nephrotic syndrome. The patient developed severe pneumonia, rhabdomyolysis, and soft tissue infection after receiving immunosuppressive therapy. He did not respond well to empiric antibiotics and was eventually transferred to the medical intensive care unit because of an acute respiratory failure and septic shock. The patient then underwent a comprehensive conventional microbiological screening in bronchoalveolar lavage fluid (BALF) and blood, and the results were all negative. As a last resort, mNGS of blood was performed. Extracellular cell-free and intracellular DNA fragments of *Legionella* were detected in plasma and blood cell layer by mNGS, respectively. Subsequent positive results of polymerase chain reaction for *Legionella* in BALF and soft tissue specimens confirmed the diagnosis of disseminated Legionnaires’ disease involving the lungs, soft tissue, and blood stream. The patient’s condition improved promptly after a combination therapy of azithromycin and moxifloxacin. He was soon extubated and discharged from ICU with good recovery.

**Conclusion:**

Early recognition and diagnosis of disseminated Legionnaires’ disease is challenging. The emergence and innovation of mNGS of blood has the potential to address this difficult clinical issue.

## Background

*Legionella* are recognized as a common cause of community- acquired pneumonia, while a rare pathogen of hospital-acquired pneumonia (HAP). Old age, underlying debilitating conditions, and immunocompromised status are risk factors for Legionnaires’ disease. *Legionella* species are best known for causing pneumonia and can also cause a wide range of extra-pulmonary manifestations, which is known as disseminated Legionnaires’ disease (DLD). Life-threatening multiple organ dysfunction can occur in severe cases (1). The diagnosis of DLD can be challenging due to the rarity of the infection and the fastidious growth in unbiased-culture based testing (2). Metagenomic next-generation sequencing (mNGS) is a nucleic acid sequencing technique with high-throughput capacity for the detection of pathogens in a single assay. A chief advantage of mNGS is unbiased sampling, which enables broad identification of known as well as unexpected pathogens or even the discovery of new organisms (3). Here, we present a case of disseminated Legionnaires’ disease in a patient with immunodeficiency disease and treated with immunosuppressive therapy, whose conventional microbiologic testing were all negative and finally achieved the correct diagnosis by mNGS of blood. This is the first case of DLD diagnosed by mNGS to our knowledge. This case warrants the attention of Legionnaires’ disease in hospitalized patients and highlights the value of mNGS technology in diagnosing the disease.

## Case report

A 52-year-old male was admitted to the department of nephrology in our hospital presenting with edema of the eyelids and bilateral lower extremities for 1 month. Laboratory findings upon admission revealed a large amount of proteinuria, hypoalbuminemia, and hyperlipidemia, which suggested the diagnosis of nephrotic syndrome. The patient then received oral methylprednisolone 60 mg daily. Meanwhile, he was diagnosed with Good’s syndrome for concurrent thymoma and significant hypogammaglobulinemia. Two weeks later, the patient developed high fever, productive cough with non-purulent sputum. Pneumonia was confirmed by chest CT, and he received empirical ceftazidime, imipenem, and intravenous immunoglobulin without good clinical response. He also complained of myalgia and muscle swelling in his left lower extremity. Twenty-two days after admission, the patient was transferred into the medical intensive care unit (MICU) because of the aggravating respiratory failure. The patient was a non-smoker and had no history of diabetes or alcoholism. He did not recall any exposure to potentially contaminated water or animals.

**FIGURE 1:**
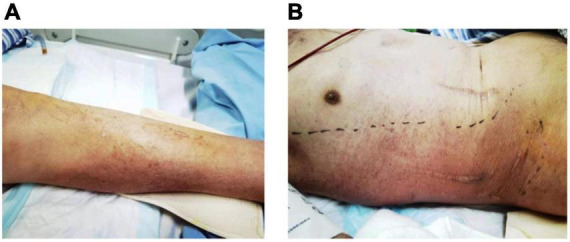
Skin congestion and swelling on the left calf **(A)** on admission, and right chest wall **(B)** 5 days after admission.

**FIGURE 2:**
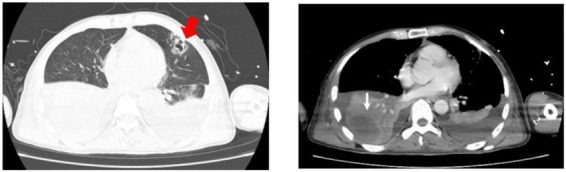
Chest CT on admission revealed patchy shadows and consolidations in both lungs and pleural effusion bilaterally. Presumed abscess in the right lower lobe (white fine arrow) and cavitation in the left upper lobe (red thick arrow) were noted.

On admission to MICU (day 0), he was drowsy and distressful. His vital signs were as follows: body temperature 38.0°C, pulse rate 125 beats/min, respiratory rate 38 breaths/min, blood pressure 116/74 mmHg, and pulse oxygen saturation 97% with a non-rebreather mask. Diminished breath sounds in the right lower lung were heard on auscultation. There was no audible cardiac murmur. His abdomen was soft and non-tender without hepatosplenomegaly. Shifting dullness was positive, along with moderate pitting edema of the limbs and lumbosacral area. The skin over his left calf was congestive and swollen with tenderness (Figure 1A). Laboratory findings upon admission revealed a white blood cell count of 2.67 × 10^9^/L with an elevated neutrophil ratio of 96.3%, hemoglobin of 120 g/L, and platelet count of 62 × 10^9^/L. The serum biochemistry panel was remarkable for striking elevation of muscle enzyme spectrum on MICU day 0, including creatine kinase increased from 10,759 to 13,514 U/L, myoglobin from 2,820 to 93,484 μg/L, alanine aminotransferase 131 U/L, aspartate aminotransferase 254 U/L and lactic dehydrogenase 1,489 U/L. The serum creatinine was 226 μmol/L with hyperkalemia. The concentration of C-reactive protein and procalcitonin was 271.33 and 100 ng/mL. Cytomegalovirus (CMV) DNA in peripheral blood was 300,000 copies/ml detected by polymerase reaction (PCR). Chest CT revealed patchy shadows and consolidations in both lungs and pleural effusion bilaterally. Presumed abscess in the right lower lobe and cavitation in the left upper lobe were noted as well (Figure 2).

After MICU admission, intravenous vancomycin and ganciclovir were added for the presumed skin and soft tissue infections and cytomegalovirus viremia, respectively. However, the patient’s condition kept deteriorating with persistent fever, skin lesion expanding (Figure 1B), rhabdomyolysis and subsequently acute respiratory distress syndrome. The patient was intubated and on invasive mechanical ventilation on MICU day 1, and continuous renal replacement therapy was initiated on MICU day 2.

**FIGURE 3:**
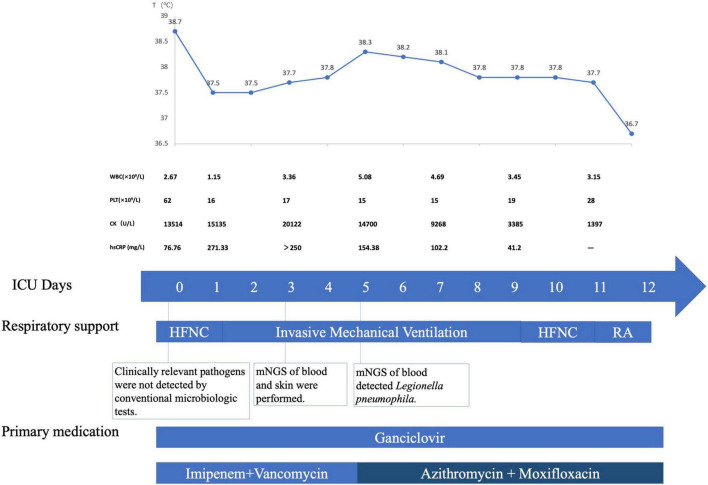
Timeline of the patient with disseminated Legionnaires’ disease. The patient was transferred into MICU on May 12, 2021, which was ICU Day 0. Major events during the course of the patient’s illness are indicated in the graph. The upper graph shows the body-temperature curve (blue line). Laboratory values obtained and primary medications administered during the patient’s ICU hospitalization are shown below. HFNC denotes high-flow nasal cannula, MICU medical intensive care unit, RA room air, mNGS metagenome Next-Generation Sequencing.

Tracheal aspiration was found to be non-purulent after intubation. The patient underwent a standard of care microbiologic diagnostics for bacteria, viruses, and fungi, including staining and culture, multiplex PCR, and serologic testing. Unfortunately, any clinical relevant pathogens were undetected. Although no pathogen was identified through comprehensive conventional microbiologic workup, infection was still highly suspected, and thus, blood and the skin specimen were sent for mNGS assay on MICU day 3. We performed mNGS on the Illumina platform by using DNA extracted from the peripheral blood and the skin specimen. In terms of blood mNGS, nucleic acids were extracted from the plasma and blood cell layer, respectively, corresponding to cell-free DNA (cfDNA) and intracellular DNA (iDNA). The total numbers of sequencing reads were 27 million, 12.5 million, and 51 million sequences for the libraries of mNGS on the plasma, blood cell layer, and skin sample, respectively. PathoXtract Nucleic Acid Kit (*WYXM03001S, Willingmed Corp., Beijing, China*) was used to extract DNA. Sequencing data were processed using Pathogen Identification Sequencing (PIseq) Metagenomic Sequencing Data Management System V2.0 (*Willingmed Corp.*) automatically. The high-quality sequencing data were compared with the human reference genome GRCH37 (hg19) by alignment software to remove the human host sequence and obtain clean data for use in the subsequent identification of pathogenic microorganisms. The clean data were aligned with the established reference database of pathogenic microorganisms to perform the annotation of pathogenic microorganism species, complete the final analysis, and obtain results on microorganism identification. We got the detection report the next day, revealing cell-free DNA (cfDNA) of *Legionella pneumophila* as high as 84,930 reads per Million (RPM) in the plasma layer, and intracellular DNA (iDNA) of the same pathogen as 6,470 RPM in the blood cell layer. mNGS of the skin specimen of left lower limb identified *Legionella pneumophila* with 349 RPM. The test also revealed low to moderate levels of CMV in both plasma and blood cell layer. Meanwhile, low level of *Pseudomonas aeruginosa* was also detected in plasma, which was 30 RPM. Further pertinent investigation revealed positive serum IgM, IgG antibodies of *Legionella*. PCR of *Legionella pneumophila* on BALF was also positive. Specific DNA sequences of *Legionella pneumophila* were identified by PCR in the skin specimen as well. On MICU day 5, azithromycin and moxifloxacin were used instead of imipenem according to the diagnosis of disseminated *Legionella* infection. The patient’s fever subsided soon afterward, along with skin alleviation of congestion and tenderness. After another 1 week of treatment, his condition improved dramatically with muscle enzymes dropping into the normal range and with the recovery of renal function. He was extubated on MICU day 9 and was transferred to general ward on MICU day 12 (Figure 3). The patient was in good condition during the follow-up. After rehabilitation exercises, the patient could take care of himself, with normal body temperature and no need of any oxygen support before he was discharged from the hospital.

## Discussion

We here reported a rare case of hospital-acquired disseminated Legionnaires’ disease in an immunocompromised patient, who had a history of Good’s syndrome and nephrotic syndrome and received immunosuppressive therapy due to nephrotic syndrome. Clinical and imaging examination demonstrated evidences of severe pneumonia, rhabdomyolysis, and local skin infection. All conventional microbiologic tests were negative, and the causative pathogen (*Legionella pneumophila*) was finally identified by mNGS.

Generally, *Legionella* was not considered on the list of candidate pathogens of HAP, therefore the diagnosis and treatment of hospital-acquired Legionnaires’ disease is often delayed. Disseminated Legionnaires’ disease is even more difficult to diagnose for its rarity and lack of efficient testing methods. There are some differences between hospital-acquired and community-acquired Legionnaires’ disease. Community-acquired Legionnaires’ disease is often caused by *Legionella pneumophila* (Lp), which often occurs in people with competent immunity, presenting non-specific clinical manifestations (1). Hospital-acquired Legionnaires’ disease is more likely to be caused by other *Legionella* species and often occurs in patients with immunodeficiency (4). Severe cases are more common in hospital acquired cases, with prone pulmonary cavities, extrapulmonary presentations such as skin eruption, myositis, pericarditis, and myocarditis, thus with a higher mortality rate (5, 6). Skin presentations are uncommon in Legionnaires’ disease and hard to diagnose, erythema, nodules, and blisters can be seen locally, while skin pathology lacks specificity (2, 7). Dagan et al. found that the mortality of HAP caused by *Legionella* is higher than CAP caused by the same pathogen, which may be attributed to the former’s delayed diagnosis and treatment as was also seen in this case (8). In the present case, the nucleic acid of *Legionella* in peripheral blood was firstly detected by mNGS. Thereafter, the detection of nucleic acid of *Legionella* in both BALF and skin by PCR further confirmed the diagnosis. Serum IgM and IgG antibodies of *Legionella* were also positive. The etiologic diagnosis of disseminated Legionnaires’ disease was made according to above clinical data in 5 days. This patient had a significantly large area of consolidation in the right lung combined with rhabdomyolysis and skin lesions in the early stage, which all resolved after azithromycin and moxifloxacin treatments specific to *Legionella.* Early diagnosis played a tremendous role in the successful treatment.

The mNGS results in this case is a turning point in the diagnosis of DLD. mNGS is a nucleic acid sequencing technique with high-throughput capacity and un-biased pathogen detection in a single assay. It has been increasingly applied in kinds of infectious diseases for its ability to discover new or unexpected organisms (3). For suspected pneumonia in critically ill immunocompromised patients, BALF mNGS and conventional microbiological tests had comparable diagnostic accuracy for bacterial and viral infections (9). For septic patients in ICU, plasma mNGS was more sensitive than blood culture in detecting bacterial infections and allowed for simultaneous detection of viral pathogens (10). mNGS show more priority in areas where conventional diagnostic approaches have limitations. At present, missed diagnosis of *Legionella* infection is still common due to limited detection methods such as gram stain and immunofluorescence stain. As for other methods, the urine antigen of *Legionella* is limited to the Lp1 serotype, and it is difficult to make the diagnosis of acute infection based on positive serum antibodies. The blood culture of *Legionella* is not sensitive because strict bacterial growth conditions are needed (1). The emergence of mNGS has the potential to facilitate the early recognition and diagnosis of *Legionella* disease, which is approved again by this case. Fast and unbiased, the technical advantages of mNGS prevent rare but lethal pathogens such as *Legionella* from being omitted in the diagnostic process and thus benefit critically ill patients. In conclusion, mNGS performs well in detecting uncommon, novel, and co-infecting pathogens without the need for *a priori* knowledge, thus providing new diagnostic clues for difficult-to-diagnose infections in critically ill or immunocompromised patients. However, it is worth noting that mNGS has some potential drawbacks and unresolved issues in clinical practice. First, mNGS is not standardized between laboratories, and interlaboratory variability makes results not comparable between laboratories. Secondly, it is challenging to discriminate causative pathogens from others (normal microbes and environmental contaminants) due to the lack of a unified approach to interpreting the result of mNGS (3). Last, as with all nucleic acid assays, the identification of microbes in mNGS does not directly confirm the presence of viable, live organisms. The clinical significance of organisms should be determined by a combination of the clinical manifestation, conventional testing, and the application of antibiotics.

Nowadays, plasma mNGS assay for identifying microbial cfDNA sequencing to predict bloodstream infection is increasingly used in critical patients. It has many limitations though, such as interference by both human nucleic acid signal and background microbial signals (11, 12). The detection of circulating microbial cfDNA in plasma presents either true bloodstream infection or circulating microbial DNA in the bloodstream derived from other local infection sources. By limiting detection to plasma, intact or intracellular microorganisms might also be missed (13). With the maturation of human-derived host nucleic acid removal technology in peripheral blood samples by PathoXtract Nucleic Acid Kit (*WYXM03001S, Willingmed Corp., Beijing, China*), the derived mNGS technology can detect DNA sequences in both plasma (cfDNA) and blood cells (intracellular DNA, iDNA). The cfDNA contains information about cells that are lysed hours or days previously, while the iDNA essentially indicates the existence of potentially alive or intact bacteria (14). Technically speaking, the detection of microbial iDNA in the blood cell layer might indicate true bloodstream infection, rather than local infection sources. Intracellular pathogens, such as *Legionella* and *Listeria monocytogenes*, can also be detected from microbial iDNA sequencing. *Legionella* sequences were detected in both plasma and blood cell layer in our patient, which suggested that *Legionella* was disseminated by the bloodstream.

Azithromycin, doxycycline, or levofloxacin can be considered as first-line therapy. β-lactams and aminoglycosides are ineffective. The combination of azithromycin and fluoroquinolones has been used in mostly severe unresponsive disease. However, there is no convincing evidence of its effectiveness (1). Early adequate therapy can reduce mortality (15). Immunocompromised patients with Legionnaires’ disease are at risk for both severe infection and relapse. In addition, extrapulmonary infections often occur in immunocompromised patients. An extended course for more than 14 days is recommended for patients with immunosuppression. The total course should be adjusted based on clinical response. Because of the risk of relapse, we also consider reducing immunosuppression when possible. If prolonged and high levels of immunosuppression are required, a suppressive course of therapy (e.g., 3–6 months) can be given (16). Our patient received 10 days combination of azithromycin and levofloxacin, reducing to azithromycin alone for 2 months. He responded promptly to treatment and has not relapsed.

Hospital-acquired Legionnaires’ disease mainly occurs in immunosuppressed patients, which results in high morbidity and mortality. This unique case indicates that mNGS is a promising unbiased diagnostic technique for early detection of *Legionella* and other unexpected pathogens. Early adequate therapy can improve outcomes of critically ill patients with DLD.

## Data availability statement

The raw data supporting the conclusions of this article will be made available by the authors, without undue reservation.

## Ethics statement

Written informed consent was obtained from the individual(s) for the publication of any potentially identifiable images or data included in this article.

## Author contributions

SL and WJ carried out the literature search and drafted the first draft of the manuscript. LW and C-YW treated the patient and gave advices. J-MP and BD were responsible for designing and revised the draft. All authors read and approved the final manuscript.
